# Innovation in Bereavement Care: Research Circles as a Framework for Translation of Research-Based Knowledge

**DOI:** 10.1177/00302228231206737

**Published:** 2023-10-18

**Authors:** Birthe Møgster, Lillian Bruland Selseng, Monika Alvestad Reime

**Affiliations:** 1 Department of Welfare and Participation, 366044 Western Norway University of Applied Sciences Faculty of Health and Social Sciences, Bergen, Norway; 2 366044 Western Norway University of Applied Sciences Faculty of Health and Social Sciences, Sogndal, Norway

**Keywords:** bereavement care, research circles, implementation, co-created knowledge translation, drug-related deaths

## Abstract

This article aims to contribute to the research-practice gap in bereavement care by exploring Research Circles as a collaborative approach to implementation of research-based knowledge into bereavement care. Particularly the article discusses key dimensions for translating research concerning bereaved after drug-related deaths into practice-relevant knowledge, as a first step of implementation. This co-created knowledge translation took place in the first phase of a Research Circle including bereaved mothers, practitioners and researchers. Data were collected via semi-structured interviews with Research Circle participants and field notes. Data were analyzed using reflexive thematic analysis. Results showed two key dimensions with the Research Circle approach influencing the translation process: (1) multiple and long-lasting arenas for translation: (2) multiple stakeholders and perspectives. Research Circles appear to be a promising framework for translating research-based knowledge in bereavement care, but there are some barriers to fully realizing the democratic ideal that underlines the Research Circle framework.

## Introduction

An average of 280 people die annually from drug use in Norway (Norwegian Directorate of Health, 2023). Drug-related deaths (DRDs) are either those caused directly by the intake of substances classed as narcotics, or those linked to drug use, such as deaths from violence, suicide, infectious disease or other health disorders (Norwegian Directorate of Health, 2014). While the mortality rate for drug-related deaths in Norway was 6.0 in 2022 (Norwegian Directorate of Heath, 2023), in Europe, the mortality rate for overdoses in 2020 was 7.4 deaths per million for the adult population. This figure is higher when other DRDs are included (European Monitoring Centre for Drugs and Drug Addiction (EMCDDA), 2022). The United States has seen an increasing epidemic of overdose deaths, with a proportion of 34.3 per 100 000 in 2021 (Centers for Disease Control and Prevention, 2022).

Each DRD leaves around 10 to 15 bereaved people (Reime et al., 2022). A DRD is an unnatural and sudden death, which can have serious consequences for those bereaved in this manner, including adverse health outcomes (Løseth et al., 2023) and a complicated grief processes (Lambert et al., 2022; Titlestad et al., 2020; Titlestad & Dyregrov, 2022) and premature death (Christiansen et al., 2020). Both bereaved parents and other relatives are found to be at risk of high levels of symptoms of prolonged grief disorder (Titlestad et al., 2021; Titlestad & Dyregrov, 2022), the most common form of complicated grief (Djelantik et al., 2020). It is crucial to support those bereaved by DRDs to prevent the extensive social and health-related challenges that may arise. However, there are significant shortcomings in bereavement care internationally (Kalsås et al., 2023; Morris et al., 2021; Schlosser & Hoffer, 2022; Templeton et al., 2017; Titlestad et al., 2020). For example, although Norway has legislation that gives those bereaved by sudden death the right to receive help, studies show that there are key organizational, knowledge-related and financial barriers in delivering the necessary support (Reime & Dyregrov, 2022; Løseth et al., 2022).

To improve DRD bereavement services in Norway, an action-promotion project a Research Circle was undertaken, in which researchers, practitioners and those bereaved by DRDs collaborated to share, adapt and implement research-based knowledge for service development. To increase knowledge about how services for the bereaved can be improved, the present article will share insights into the knowledge provided by the work about implementation and, more particularly, the process of knowledge translation. While implementation refers to the process of putting a new policy into effect (Howlett et al., 2020), knowledge translation describes the process prior to implementation, based on the assumption that new knowledge must be translated and adapted to the specific implementation context (Røvik, 2007). One barrier identified in bereavement care innovation is the lack of development of “formal knowledge exchange processes between grief and bereavement care researchers and practitioners” (Hay et al., 2021, p. 338). Therefore, interventions might not be targeted, feasible and applicable (Hay et al., 2021). In recent years, policymakers and researchers have become increasingly interested in how to adapt knowledge and ideas to local contexts, including involving service users and practitioners in public sector innovation (Osborne, 2018; Osborne et al., 2016; Torfing et al., 2019). Osborne (2018, p. 225) defines co-creation as a process including public service organizations, citizens and service users, which “assumes an interactive and dynamic relationship where value is created at the nexus of interaction.” The concept of co-created translation developed for framing the translation process in the present article relies on co-creation aspects. Breen & Moullin (2022) point to a problem in innovation in bereavement care, in which context-specific investigation of specific evidence needs to be translated. Identifying facilitators and barriers to the application of this evidence in practice is often missing, particularly from the perspectives of people who provide bereavement care. Therefore, there is a need to explore and develop models that can facilitate collaborative, context-specific implementation of research-based knowledge into practice-relevant knowledge relevant to bereavement services.

The present article explores key dimensions for knowledge translation from the first phase of a Research Circle initiated to implement research-based knowledge on DRD bereavement into practice.

### The Research Circle Approach

The Research Circle approach relies on democratic and emancipatory ideals inspired by the Swedish education tradition of “study circles” (Holmstrand et al., 2008). Research Circles are phase-based collaborations, lasting between one and two years, in which researchers and practitioners meet to immerse themselves in a topic of common interest (Holmstrand et al., 2008; Persson, 2016). More recently, service users have also been included in Research Circle collaborations (Follevåg & Seim, 2021). Research Circles consists of three phases: (1) systematically working to identify service challenges, gaining relevant knowledge; (2) testing out and (3) evaluating different measures (Persson, 2016). Research Circle work is characterized by being experimental and innovative, in which participants (including the facilitators) work in partnership as equal collaborators (Holmstrand et al., 2008; Persson, 2016; Löfqvist et al., 2019). Positions between participants are said to be “unlocked”; pre-defined roles are set aside and participants collaborate to explore a theme or a challenge (Persson, 2016, p. 160)*.* The innovative aspect to the approach is how participants work with a set of knowledge. All participants immerse themselves in a collaborative knowledge development process in which they can challenge, modify and develop new knowledge (Persson, 2016). As a result, participants can gain increased knowledge and understanding of the topic, and new perspectives and changes can be addressed (Persson, 2016).

To the best of our knowledge, Research Circles have thus far not been used to translate and implement new research-based knowledge. The present study therefore makes two contributions: first, it adds to knowledge on the use of Research Circles as an approach for research-based knowledge translation; second, it adds to knowledge about innovation in bereavement care.

### Theoretical Framework

As a theoretical frame, the authors will apply Røvik’s (2007) theory of translation, which develops translation theory from its origins in linguistic studies and the translation of texts into organization theory. He poses an instrumental framework for studying the travel of practices and ideas between organizations, “developing knowledge about how to conduct translations of practices and ideas to achieve various organizational ends in knowledge transfers” (Røvik, 2016, p. 292). The authors believe that the translation of research-based knowledge has much in common with the translation of practices and ideas and, as such, Røvik’s framework offers a useful lens for enhancing our understanding of research translation in the Research Circle.

Røvik (2007) describes the knowledge translation process in two analytically separate stages. First, ideas must be decontextualized (translated from its origin context) and second, recontextualized (translated into) to a new practice and organizational context. Practices and ideas can be translated by copying, addition, omission or alteration (Røvik, 2016). The characteristics of practices and ideas can influence the outcome of the translation process, as they can be explicitly formulated or more abstract. Røvik (2016) refers to Polanyi’s (1966, p. 4) concept of tacit knowledge, which describes the knowledge that is difficult to express orally—“we can know more than we can tell”. Practices and ideas can also be complex (e.g., the clarity of relationships between cause and effect). Finally, practices and ideas can be embedded within a particular organizational context which can influence the extent to which they are transferable to other contexts. Arenas where ideas move between organizations, such as meetings, seminars, classes or conferences, also have different features but are essential in the outcome of the translation process (Røvik, 2007).

Røvik (2016) highlights the importance of the translator’s competence, e.g., whether the translator has inside knowledge of the ideas and practices to be translated, or of the field more generally. Translation competence is the “ability of translators to translate practices and ideas between organizational contexts in ways that increase the probability of achieving organizational ends” (Røvik, 2016, p. 299). It is multi-faceted, as the translator(s) should have in-depth expertise about the practice or idea and contextual knowledge about both its source and about the recipient (Røvik, 2007). Røvik (2007) uses the concept of “outbringer” to refer to people with this knowledge. The translator’s position also influences the translation process (e.g., their status within the field, whether they are a manager, their level of education, etc.) (Røvik, 2007). New ideas are often abstract or internal and can be difficult to relate to a new context; this is also the case with research-based knowledge. As such, a translation phase is needed before new ideas can be put into effect (Røvik, 2007).

### Translation of Research-based Knowledge from the END Project

The knowledge translation process studied in the present article derived from the Drug-Death Related Bereavement and Recovery Study (the END project; https://www.hvl.no/end), conducted between 2021 and 2022. The END project aimed to improve the lives of bereaved family members and friends after a DRD by studying their perspectives, health, functioning and need for bereavement care. The Research Circle to translate and implement new research-based knowledge from the END project was divided into three phases: (1) the knowledge translation phase, in which participants were presented with research-based knowledge from the project directly by the researchers (outbringers); (2) the implementation phase, in which participants put this knowledge into action by choosing an innovation project suitable for bereavement care and support needs; (3) the evaluation phase, focusing on both local innovation projects and the entire body of work of the Research Circle.

The present article focuses on the first phase, which consisted of gaining relevant knowledge, identifying service challenges and translating research-based knowledge into practice-relevant knowledge for use in bereavement care. This phase lasted from August 2021 to March 2022 and comprised six whole-day meetings, two of which lasted over two days, with a total time of 46.5 hours. Research Circle gatherings were conducted in two sequences, separated by a lunch break. Both sequences started with 20–45 min presentations from one of the researchers from the END project on a scientific-based theme. There were then reflection rounds, where all participants were offered space to discuss the presentation. Relevant research articles were sent to participants in advance. Participants prepared their local innovation projects during this phase and had “homework” to plan progress tasks between meetings (see Table 1).

**Table 1.** Research Circle Phase one: Meetings and Themes.

Meeting	Date	Time spent (both formal and informal)	Participants	Theme (s)
1	Aug 2021 – two days of initial gathering	Formal work: 7 hours Informal gatherings (lunch and dinner): 4 hours	12	Presentation of the END project and Research Circle approach Key questions: How do we organize ourselves (including creating functional processes in the Research Circle), and what outcome do we want to create? What knowledge do we want from the END project?
2	Oct 2021	Formal work: 4.5 hours Informal gathering (morning coffee and lunch): 1.5 hours	10	Evaluation of meeting 1 Anchoring in own organization Theme 1 from END: Before death and parental grief Reflections on theme 1 Theme 2 from END: How do parents adapt to the risk of complicated grief? Reflections on theme 2 Summary discussion and evaluations
3	Nov 2021	Formal work: 4.5 hours Informal gathering (morning coffee and lunch): 1.5 hours	10	Evaluation of meeting 2 Anchoring – obstacles and facilitators Theme 3 from END: Stigma Reflections on theme 3 Theme 4: Crisis guidelines Reflections on theme 4 Homework: Implementation, anchoring support and barriers
4	Dec 2022	Formal work: 4.5 hours Informal gathering (morning coffee and lunch): 1.5 hours	8	Evaluation of meeting 3 Anchoring and implementation (homework) Theme 5 from END: Grief theory and processing grief Reflections on theme 5 Theme 6 from END: How to reduce stigma of bereaved people and reduce stigma in society Reflections on theme 6 and how to use this knowledge Implementation and anchoring (lecture) Evaluation
5	Feb 2022	Formal work: 4.5 hours Informal gathering (morning coffee and lunch): 1.5 hours	9	Evaluation of meeting 4 Theme 7: Peer support Reflections on theme 7 Presentation of planned local innovation projects Reflections on planned local innovation projects Summary discussion and evaluation
6	Mar 2022 – two days of gathering	Formal work: 7.5 hours Informal gathering (lunches and dinner): 4 hours	10	Theme 8 from END: Own substance use challenges among those bereaved by DRD Reflections on theme 8 Theme 9 from END: Friends as bereaved Group work and presentation of planned local innovation projects Evaluation gathering; knowledge translation process and phase one of Research Circle
Total		Formal work: 32.5 hours Informal gathering: 14 hours Total: 46.5 hours		Nine themes. Seven include scientific research from END, two include topics relevant to bereavement care

### Participants

The Research Circle involved 11 participants: two mothers who had lost a child to a DRD, four practitioners from municipalities and Non-Governmental Organizations (NGOs) providing health and welfare services, two educators/researchers from an educational institution and two researchers from the END project who facilitated the Research Circle, from the same academic institution (authors one and three). One of the municipality practitioners was replaced during phase one due to a lack of time to participate. Participants were recruited via the END project group and advisory board. All participants were women with substantial experience of working either with drug addiction and mental health or grief. Nine participants had experience and knowledge about DRDs through personal experience, research or practice.

## Methods

Individual interviews were used to explore participants’ experiences of the knowledge translation process performed in the first phase of the Research Circle. Eight participants in the Research Circle (all except the leader, co-leader and one participant who had left the group) were invited and consented to participate.

### Data Collection

Interviews were conducted in April and May 2022, after phase one of the Research Circle. Interviews occurred at a location of the participant’s choosing (e.g., at home or in their office), lasting between 60-90 minutes and focusing on the following topics about participation in the Research Circle: prior expectations; experiences of participating in the first phase; experiences of the knowledge presentations; experiences of new knowledge; experiences of barriers and facilitators to the translation of knowledge; use of the knowledge gained. Interviews were audio recorded and transcribed. Supplementary field notes consisting of observations by the first author and transcribed audio recordings of discussions between the first and third author after each meeting were also included. These data contained descriptions of how the meeting was conducted, major events and reflections on the processes in the meeting. Field notes also included frames and content of the Research Circle (see Table 1).

### Data Analysis

Reflexive Thematic Analysis (RTA; Braun & Clarke, 2021) was used to identify codes and patterns (themes) about what hindered and facilitated the knowledge translation process. The authors followed the six phases of RTA. First, we familiarized ourselves with the transcriptions by reading and re-reading the text (1). Then, the first and third authors systematically coded the dataset into two separate documents based on the research question: *What facilitates and hinders knowledge translation in Research Circles?* The interview text was coded separately before comparison to avoid researcher bias (2). There were significant similarities in the authors’ two separate coding documents and together, all three authors generated initial themes from the coded dataset (3), developed and refined themes (4) and reviewed and named the themes (5). The first author started writing up the research (6) and completed it in collaboration with the other authors.

The authors’ background as social workers (LBS and MAR) and the first author’s background as a social educator influenced the research process, including formulating research questions, developing interview guides, the analysis process and the write-up. All authors were also members of the END project. The role of first author (co-facilitator) and third author (facilitator) in the Research Circle is also relevant. Their presence during the Research Circle meetings allows them to recognize nuances within the interviews, but their participation might also influence the choice of interview questions and analysis. The first author, co-facilitator and Research Circle participant conducted the interviews and this triple role may have influenced participants’ responses.

Gough & Madill (2012, p. 374) suggest that researchers have a “reflexive scientific attitude,” where their subjectivity is seen as a primary tool and a resource when analyzing data. The authors also adopted a reflexive scientific attitude, as the authors shared their developing analysis to obtain participant feedback. The first and third authors performed a member check validation by presenting analyzed data to the Research Circle participants (Birt et al., 2016; Harvey, 2015), who largely acknowledged and confirmed the findings. This validation also influenced the presentation of results and discussion, as participants suggested the need for emphasis on the time-consuming and process-oriented way of working in Research Circles. Authors were also invited to research team meetings in between Research Circle gatherings.

### Ethical Considerations

The END project and the Research Circle study were approved by the Norwegian Regional Committees for Medical and Health Research Ethics (2017/2486/REK vest) and the Norwegian Social Science Data Service (NSD-52,551). Before the Research Circle started, all participants were informed about the study and about their rights as research participants (following the Declaration of Helsinki) and gave written consent. In the following presentation of results, all participants are given pseudonyms and any other identifiable information has been redacted.

## Results

The analysis identified two key dimensions of knowledge translation: (1) multiple and long-lasting arenas for translation; (2) multiple stakeholders and perspectives. The key dimensions further contain codes reflecting the meaning units from the participants.

### Multiple and Long-lasting Arenas for Translation

Participants highlighted the value of accessing various arenas to share, discuss and reflect on relevant knowledge. These arenas included dissemination of the research topic, reflection rounds in the plenary and more informal moments for reflection such as breaks and social gatherings. They also highlighted that it was essential that the conversations about knowledge were with the same participants over a limited but continuous time.

#### Variety of Reflective Arenas

Formal and informal arenas were both seen as essential facilitators for reflection with others. In formal reflection arenas, all participants were encouraged to contribute to reflection rounds after the researchers presented a research topic. Most participants stated that they appreciated giving their thoughts about the topic presented, as exemplified by Karin:

Because something happens to you when you sit and listen to others (…). There will be a round of reflection that will be so immediate, and then there will be someone else who may connect to what has already been said. And that’s nice, there will be more layers then. (Karin)

In the reflection rounds of the first phase, participants connected what was presented by researchers (research-based knowledge) to their prior knowledge and experiences from the field of practice. Informal arenas were often used to elaborate and reflect with other participants on the presented knowledge:

And then it’s exciting when you get to present something, there’s a round of reflection, and then we sit and eat lunch together, and then you start talking about it, and then suddenly, “oh yes, I understood it during the presentation in this way, but then it has gone on to the third step, so maybe I understand it a little differently,” and look at what you, as a bereaved person, for example, is telling me about what we have seen two steps before. So, it’s an ongoing process, I think. (Frida)

In this quote, Frida describes an ongoing process in various formal and informal reflection arenas in which she translates knowledge about various research topics step-by-step in conversations with other participants.

#### Continuity

Participants emphasized the importance of the Research Circle’s collaborative way of working. They pointed to the process of becoming a stable group of people in recurring meetings, first over six months and then with a further year’s continuation, as important for creating a trusting atmosphere that facilitated open reflection. Participants described the development of a group climate that facilitated more personal involvement. In addition, insight into the topic’s relevance also developed over time. For example, one participant, Janne, had lost a child to DRD and, at the beginning, she did not think she needed any more knowledge. However, she described a process in which she became more open-minded:

In a way, you don’t need all that knowledge either, as long as you are bereaved (…). I think when you are a bereaved person, then you have the whole picture. In a way, we know everything about that grief and what it entails (…). And then you learn and see this research presented. Then, in addition, you also get to hear how others are doing. I have heard that before, but we get to hear a bit more about research that may be relevant as well. Because suddenly, someone appears who has a slightly different background and skills. (Janne)

The diversity of participants’ knowledge about DRDs when they joined the Research Circle was also found to influence the knowledge translation process. For Karin, the opportunity to prepare and read in advance was an essential facilitator:

So, I have really enjoyed these articles and actually taking the time to read them beforehand has been a helpful and good way for me (…). I have had a completely different focus than if it had been completely new (…). I have listened differently because I have read beforehand. (Karin)

She said that she did not know much about the field when she joined the Research Circle. However, preparatory reading of the articles gave her advanced knowledge so she could more easily translate the presentations into her practice context.

Access to multiple arenas over a continuous period together with the same participants resulted in all participants experiencing the translation of research-based knowledge in ways that could be adjusted to their prior knowledge base. This, in turn, offered them a step-by-step translation of the topics presented in the first phase of the Research Circle.

### Multiple Stakeholders and Perspectives

Participants occupied different roles, as researchers, as people bereaved by DRD and as professional workers in different institutional contexts. Some held multiple positions, e.g., professionals who themselves had been bereaved. This group diversity interacted with the translation process in several ways, as it offered participants access to different perspectives and experiences, which gave them new ways of seeing and understanding. However, the diversity also led to some challenges.

#### Personal Motivation and External Commitment

The level of personal and professional engagement in bereavement care was an essential facilitator for knowledge translation, e.g., researcher on the topic or as bereaved. Several participants had experienced losing a family member or a close friend to a DRD or, if not, were able to relate to the loss of family members from other causes. Death and bereavement are sensitive topics that can be challenging to address. However, in the Research Circle, these topics also became a central dimension that generated recognition and a sense of unity. The link to personal experiences seemed to increase participants’ personal motivation and external commitment, as Trude describes:

And suddenly you go into yourself a little, and then you think about those you have lost and then you think about things that have been difficult or you have been frustrated with (…). It also gives extra motivation, an extra inner motivation to be able to do something for these people, because it suddenly becomes very important, because you suddenly realize that this is actually also a bit personal. (Trude)

Trude came to realize that she had more experience with bereavement after DRD than she had initially thought, both as a secondary victim (loss of clients at work) and in her personal network. As a result, she gained personal motivation to contribute to improving bereavement care.

All participants had to work within the parameters of the local context of the particular bereavement care innovation project that they planned to establish in their own organization. Most professionals had significant experience in working with people bereaved by DRDs and held assumptions about the help and support that people would need. When the research-based knowledge from the END project confirmed their experience-based knowledge, it engaged and motivated them by increasing their opportunities to share what they already knew:

One thing is what you think of yourself as a professional, but that doesn’t help because if it hasn’t been researched, it doesn’t apply. You can think and you can discuss, but basically, you must have research to back up what you propose. (Frida)

The quote from Frida also illustrates that participants tended to rank research-based knowledge above other forms of knowledge. They felt they needed research-based knowledge to add legitimacy to any suggestions they made for bereavement innovations, which motivated them to engage in knowledge translation processes.

#### Position and Status

Participants were motivated by the opportunity to gain access to others’ perspectives and knowledge. However, they also had perceptions of the position and status of other group members and research presenters that hindered open and explorative communication and knowledge translation. For example, several participants had a preconception of the researcher’s position as an expert defining the truth about a topic, which inhibited the sharing of immediate thoughts and ideas about the presented knowledge. One participant felt that it was more challenging to ask questions if the presenter was an experienced, senior researcher. Another participant, Janne, reflected on her thoughts about what researchers conveyed and how she perceived their statements as truth: “*But it’s not something you sit and ask a researcher about, ‘Why is it like this?’ Because they are telling you why it is so.”* Participants’ understanding of research as “the truth” and the researcher’s position made it difficult for them to ask questions about the research itself. Experienced researchers could also confirm their positions by giving receipts for establishing help and support for people bereaved by DRDs. Ida shared her concerns about researcher status:

I was a bit worried at the time Dina was there because it was a bit about the fact that, because she has such high competence (…). I saw that many people started to ask, “give me some tips.” So that self-creativity, what is happening now? If everyone is just going to write down how we’re going to do this here. (Ida)

Many participants “took for granted” the ideas given to them by experienced researchers, especially in planning local innovation projects.

The researchers presented study findings that showed that people bereaved by DRDs often experience comments from others as hurtful and stigmatizing (see Table 1). Several participants highlighted that they did not want to be associated with this negative attitude and did not want to say anything that could harm the two bereaved mothers in the group. They had great respect for the bereaved mothers and further exploration became difficult because of the fear of hurting them. Participants were careful with asking questions or airing views that the bereaved mothers may perceive as stigmatizing and hurtful, as illustrated by Lone: “*I have been afraid of saying something wrong, especially in relation to those left behind. I have been afraid to say something that might offend.”*

Furthermore, the emotional pain associated with losing a child was difficult for others to comprehend. Field notes show that participant roles and fear of hurting one another were discussed on multiple occasions. These dimensions had an important influence on the knowledge translation process. Perceptions and preconceptions of others can hinder an open, honest and exploratory dialogue for creating new understandings and translating new knowledge into use in bereavement care.

When the topic of grief was discussed, it was presented in the context of participants’ own experiences with loss. Current or recent grief seemed to affect the knowledge translation process, as participants limited their reflections and discussion through sadness from their own loss and from fear of hurting others.

## Discussion

This study aimed to give insight into key dimensions for knowledge translation in Research Circles. Results show that there are some important characteristics of the Research Circle approach that can facilitate knowledge translation, but also some obstacles.

### An Arena to Test, Share and Discuss Research Findings

The Research Circle’s first phase provided a long-lasting and stable translation arena where participants could test, share and discuss research findings with other participants over time and in different settings. Access to several reflective arenas, both formal and informal, was an essential facilitator in ensuring that knowledge translation was an ongoing process. Røvik (2007) emphasizes the importance of outbringers establishing internal areas and time for conversations to develop and understand practices and ideas. In our Research Circle, participants highlighted the importance of informal discussions with others to develop their understanding of the presentations and adapt the key messages to their work and their planned innovations. This finding supports the ethnographic studies of Gabbay and le May (2016), where informal arenas for everyday chat, e.g., coffee rooms, are essential arenas for dialogue and story-swapping in transforming knowledge into use.

The knowledge translation process appeared to render research-based knowledge more explicit and transferable. Research or new ideas can be perceived as abstract, academic or as embedded in a different context, which can make them unfamiliar to professionals who are supposed to implement the knowledge in their own practice. Røvik (2016, p. 295) states, “the more explicit the knowledge, the easier it is to translate the knowledge”. Conversely, a practice or idea can take the form of more tacit knowledge, referred to by Polanyi (1962) as non-verbalized, non-standardized and non-codified characteristics. Our results show that spending time with the same people created a safer environment for participants to express themselves, talk deeply about research-, evidence- and practice-based knowledge, and promoted reflections and questions. The Research Circle frames created allowed participants to verbalize their tacit knowledge, which further seemed to contribute to the translation process.

### Co-created Translation

Co-created translation is the process in which diverse participants collaborate to translate new research-based knowledge into practice-relevant knowledge, by sharing their understandings of topics and synthesizing these understanding with existing practice and research-based knowledge through a continuous process. The Research Circle was conducted with bereaved mothers, practitioners and researchers who had an interest in and experience with bereavement care. This diversity ensured a variety of perspectives and knowledge. Persson (2016) describes Research Circles as a space for interpretation and translation of research-based knowledge in relation to other knowledge forms and positions. Participants perform knowledge development with one another, which requires the creation of a sharing atmosphere (Persson, 2016). The structure of the Research Circle gradually gave rise to expected frames for open dialogue and facilitated elaboration of different perspectives that made the co-created translation possible. Translation of new research-based knowledge then gradually developed in a way that adheres to Osbornes (2018) concept of value creation, highlighting the interactive and dynamic relationship in which value is created.

The diversity of perspectives brought by the participants, as well as their commitment to working with a local innovation project, appeared to gradually increase their motivation and engagement in the process. Some also reported that they developed a better understanding of the relevance of innovations in DRD bereavement care. Other studies of the Research Circle approach show similar findings, as participants experience collaboration as enriching (Bjerkholdt et al., 2017) and recognition of narratives as engaging (Fjetland et al., 2019).

Our study shows that including bereaved mothers’ and experienced practitioners’ perspectives enabled a translation process where user- and practice-based experiences were added to research-based knowledge. When creating targeted and needs-based innovations in public sector, it is important to include multiple stakeholders to develop solutions based on democratic participation and adapted to the public sector context (Torfing et al., 2019). The inclusion of service users has become widely recognized in public service innovation (Torfing et al., 2019). However, bereaved people are often excluded from bereavement service development, which can lead to a lack of targeted bereavement care for the particular service user group (Hay et al., 2021). Where they included, the contributions of bereaved people might still be limited if their views are seen as incontestable. There is also a risk of tokenism when people are included as a gesture rather than in genuine partnership (Torfing et al., 2019), which restricts the level of insight than can be gained (Persson, 2016). In our study, the continuous time spent together and clear discussion of the positions and roles of bereaved participants challenged preconceptions and created a more open dialogue. Similar findings were shown in Follevåg and Seims’ (2021) study of Research Circles as a tool for bridging the gap between patients and staff in a health institution, as patients reported that they appreciated honest and constructive feedback from staff. However, this perspective change did not occur in relation to research and researchers, who continued to be viewed as incontestable. This partly remained a barrier to open exploration of new knowledge and, as such, the democratic ideals of Research Circles’ principles and the notion of “unlocked” positions (Persson, 2016) were not fully reached.

### The Influence of Outbringers

The study shows that the outbringers’ (the researchers) role and presence in the first phase of the Research Circle influenced the translation processes. The purpose of presentations from the outbringers was to introduce new research findings from the END project for use in practice. Røvik (2007) argues that outbringers’ skills should be related to the idea (here, findings from the END project) and the audience’s personal and organizational needs. Outbringers are often only involved in the decontextualization part of the knowledge translation process by presenting new ideas and practices, e.g., at conferences, meetings, seminars or classes (Røvik, 2007). In our study, the outbringers were also present during reflections and planning of innovative services, meaning they could also contribute to the recontextualization of knowledge. Participants experienced outbringers’ engagement in discussions as important for translating research-based knowledge into practice-relevant knowledge, as they had the opportunity to consult “the experts” and their “inside” knowledge. As such, the outbringers also contributed to the co-created translation process.

In their Research Circle study, Löfqvist et al., (2019) found that participants wanted to learn about new research findings and develop new thinking about the research topics. It was necessary to adjust the level of the presentations to match the participants’ skill and knowledge levels, in order to establish “a challenging level for the discussions” and prevent those who were highly skilled from dropping out (Löfqvist et al., 2019, p. 94). At the same time, presentations that were too advanced could be challenging for those new to the field. Our findings show that several participants viewed some of the presentations more as guidelines or instruction manuals for innovation, rather than as research that needed contextual translation. This seemed particularly the case if the outbringer was a senior researcher, which demonstrates Røvik’s (2007) view that the outbringers’ position influences how recipients receive and assess the value of the information provided. Local organizational knowledge might be lost if others’ ideas and practices are replicated directly without being translated to fit the specific organizational and user needs (Røvik, 2007).

### Research Circles as a Possible Framework for Bereavement Care Innovations

This study’s key dimensions have shown that the Research Circle approach might be a useful framework for translating research-based knowledge into practice-relevant knowledge. However, there are some features of bereavement care that must be considered in order to realize the full potential of the Research Circle approach. The sensitivity of topics such as death, grief and bereavement can influence the extent to which participants share their experiences and perspectives. They can form a common frame of reference that can positively influence willingness to share, but can also inhibit expression through a fear of hurting others. Røvik’s (2016) knowledge translation theory is more instrumental and does not account for the sensitivity of the content of translated ideas and practices. However, Røvik (2007) emphasizes that outbringers’ inside knowledge can influence translation. Their position as experts in the field may convey advantages through familiarity with the field and with its particular inherent barriers to knowledge translation. In the case of sensitive topics, it is essential that the outbringers are aware of the dynamics of taboo topics and emotional sensitivity and are prepared to explicitly acknowledge this in group discussions.

Our Research Circle design highlights several steps and components of knowledge translation and implementation, including the co-created translation process in the first phase, further planning of implementation of practice-relevant knowledge in local innovations in the second phase and evaluations in the last phase. Breen & Moullin (2022, p. 642) suggest applying multiple frameworks from implementation science to address all core components of translation and implementation, including those that “guide the process of implementation, another for the selection of implementation determinants, and another for evaluation”. These core components are present during the phases of a Research Circle. However, Research Circles are also time-consuming for participants. Higher costs and the time-consuming nature of activities are often seen in collaborative approaches in the public sector (Steen et al., 2018). Hence, if barriers such as topic sensitivity and pre-established positions are not addressed, there could be a waste of time and resources and a lack of end benefit.

In summary, our study shows that the Research Circle approach’s collaborative design is a promising approach for narrowing the research-practice gap in bereavement care by providing a holistic and enduring arena for co-created translation of new research-based knowledge. This can lead to strengthening the foundation for creating practice-relevant knowledge for future innovations in bereavement care.

## Strengths and Limitations

Our study draws on several data sources and all participants in the Research Circle consented to be interviewed. The authors have also been transparent in how the study was conducted. Although the study is limited to one Research Circle, the authors believe that the findings may be transferable to other contexts where multiple stakeholders with different positions and knowledge collaborate in knowledge translation processes.
